# Venous Malformations: Unraveling Latest Mechanisms and Bridging Gaps in Targeted Therapy Development

**DOI:** 10.7150/ijbs.122569

**Published:** 2025-10-27

**Authors:** Xuan Jiang, Li Hu, Jiayi Lai, Shengfang Ge, Hui Chen, Xi Yang, Xiaoxi Lin

**Affiliations:** 1 Department of Plastic and Reconstructive Surgery, Shanghai Ninth People's Hospital, Shanghai Jiao Tong University School of Medicine, Shanghai, China.; 2 Department of Biochemistry and Molecular Biology, Shanghai Jiao Tong University, Shanghai, China.; 3 Department of Laser and Aesthetic Medicine, Shanghai Ninth People's Hospital, Shanghai Jiao Tong University School of Medicine, Shanghai, China.

**Keywords:** venous malformations, vascular anomalies, genetics, PI3K/AKT/mTOR pathway, targeted therapy

## Abstract

The vascular system plays a crucial role in maintaining homeostasis, ensuring the supply of oxygen and nutrients to tissues, while facilitating the removal of metabolic waste. Additionally, it contributes to immune defense, temperature regulation, and the transport of hormones and signaling molecules. Vascular anomaly (VA) arises due to developmental abnormalities or functional defects in the vessels. This review describes venous malformations (VM), a rare disorder predominantly caused by somatic mutations. Advances in recent research have substantially improved our understanding of the molecular mechanisms underlying these malformations, largely through the identification of their genetic origins and the study of animal models and endothelial cells derived from patients. Most of the somatic mutations associated with venous malformations affect genes within oncogenic growth factor signaling pathways, making it possible to repurpose certain cancer therapies to treat these VAs. This article summarizes the key molecular findings and explores emerging therapeutic strategies aimed at novel targets.

## Introduction

The cardiovascular system sustains human homeostasis by delivering oxygen, nutrients, and hormones while removing metabolic waste 1, 2. The vascular system-comprising arteries, veins, and capillaries-embodies blood circulation. Each vessel type has distinct structural and molecular features supporting its specific function 3. Abnormal blood vessel growth, structure, or function caused by genetic defects or damage from surgery, trauma, or radiation leads to various health problems 4. Vascular diseases include various disorders of arteries, veins, and capillaries; each has distinct mechanisms and manifestations.

VM are congenital venous disorders involving abnormal vein overgrowth, which can be localized or multifocal. They cause pain, mobility loss, organ dysfunction, and increased thrombosis risk, potentially becoming life-threatening. VMs stem from somatic mutations, causing diverse symptoms 5. Understanding VMs requires knowledge of venous channel development and its regulatory mechanisms. Massively parallel sequencing has enabled identification of the genetic causes of various VMs 6. Meanwhile, single-cell transcriptomics uncovers vascular endothelial heterogeneity across vessels/organs, providing insights into vein regulation/disease and revealing new therapeutic targets 6.

## 1. Mechanisms of Vascular Angiogenesis, Maturation, and Maintenance

The vascular lifecycle-spanning angiogenesis, maintenance, quiescence, and aging-is central to many severe diseases. Understanding vascular endothelial cells (ECs) is essential for deciphering the pathogenesis and progression of venous VMs 7.

Angiogenesis initiates with capillary sprouting, guided by vascular endothelial growth factor (VEGF) gradients 8. ECs degrade the basement membrane and migrate directionally. This coordinated sprouting is regulated by Notch/Delta signaling to maintain vascular integrity 9. Endothelial migration precedes proliferation, leading to anastomosis of sprouts and formation of vascular networks, often supported by myeloid-derived accessory cells 10. Vascular network maturation is tightly regulated by platelet-derived growth factor (PDGF) /PDGF receptor (PDGFR) signaling, angiopoietin/TIE signaling, and pericyte recruitment 11.

Following angiogenesis, ECs enter a state of quiescence, which is actively maintained rather than a default condition 12. Sustaining vascular quiescence involves a complex interplay of biochemical signals, including laminar blood flow, PDGF/PDGFR signaling, angiopoietin/TIE pathways, and pericyte recruitment 12. A key regulator in maintaining the quiescent endothelial layer is the continuous activation of the phosphoinositide 3-kinase (PI3K) / protein kinase B (AKT) / mammalian target of rapamycin (mTOR) signaling pathway, which phosphorylates forkhead box (FOX) transcription factors, leading to their exclusion from the nucleus and silencing their transcriptional activity 13.

The interaction between capillary ECs and pericytes, characterized by pericyte foot processes penetrating the basement membrane and the release of latent transforming growth factor β (TGF-β), further enhances endothelial quiescence 7. This dynamic is known as "vascular normalization", which explains why anti-VEGF/VEGF receptor (VEGFR) therapies predominantly target immature blood vessels lacking pericyte coverage while sparing mature, pericyte-associated vessels 14.

Consequently, the quiescent state of the endothelium is an active process that suppresses the transcriptional program associated with EC activation. Disruptions or mutations within this regulatory framework can lead to aberrant angiogenesis, as seen in VM.

## 2. Clinical Characteristics and Classification of Venous Malformations

VMs constitute the most prevalent type of vascular malformation, accounting for approximately 70% of all cases, with an estimated incidence of 1% 15. These slow-flow vascular lesions result from defects in vascular morphogenesis occurring during early embryonic development, typically between 4 and 10 weeks of gestation 16. Clinically, VMs are characterized by their blue, soft, and compressible appearance, predominantly affecting the skin and mucosal membranes 5. Histologically, they are distinguished by enlarged and distorted venous channels, where a single layer of ECs is surrounded by a disorganized extracellular matrix, resulting from reduced fibronectin expression and increased proteolytic activity, along with smooth muscle cells (SMCs) 17, 18. These lesions are present at birth and grow in proportion to the individual's body size, manifesting as isolated or multifocal localizations, particularly involving the face, limbs, and trunk, with the head, neck, and maxillofacial regions being the most frequently affected areas 19.

Accurate clinical classification of VMs is critical for diagnosis and treatment. While prevailing systems focus on clinical presentation rather than molecular mechanisms, molecular advances have identified causative genes, including germline mutations. This review adopts the classification framework established by the International Society for the Study of Vascular Anomalies (ISSVA), which is continually updated to reflect new insights into the biological and genetic basis of VAs.

**Figure 1:**
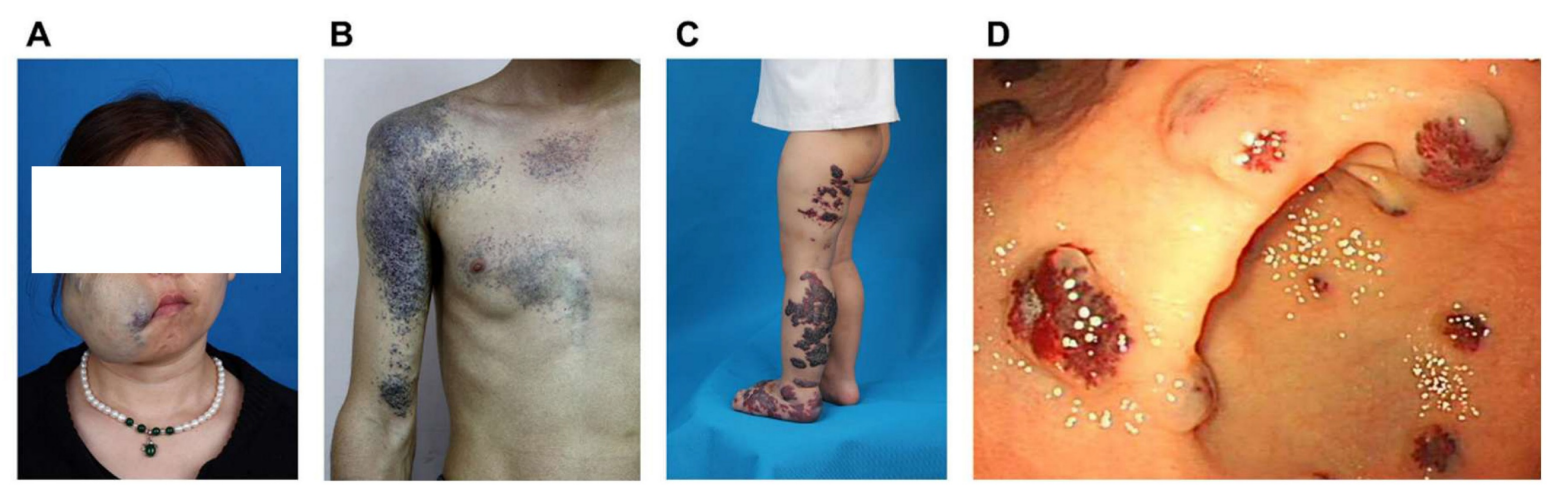
Examples of clinical characteristics of different phenotypes of venous malformations. A) Sporadic venous malformation of the maxillofacial region; B) Glomuvenous malformation of the right upper extremity and chest; C) Verrucous venous malformation of the left lower extremity and foot; D) Gastrointestinal blue rubber bleb nevus syndrome. The clinical presentation of familial venous malformation cutaneo-mucosal does not markedly differ from that of typical sporadic venous malformations.

According to the latest ISSVA criteria, VMs are categorized into several subtypes, including common sporadic VM, familial VM cutaneo-mucosal (VMCM), blue rubber bleb nevus syndrome (BRBNS), verrucous venous malformation (VVM), glomuvenous malformation (GVM), cerebral cavernous malformation (CCM), familial intraosseous vascular malformation (VMOS), among others 20. This review specifically focuses on VMs located on the body surface (Figure 1).

The clinical characteristics of common VMs have been previously detailed 5. VMCM resembles multifocal VMs and exhibits autosomal dominant inheritance. These malformations typically present as multiple lesions, often apparent at birth or in early childhood. They progressively enlarge throughout life, with characteristic involvement of facial structures-particularly oral mucosa, lips, and tongue 21.

BRBNS is marked by the presence of bluish-violet papules and nodules of varying sizes distributed across the body. These soft, rubbery, and non-tender lesions are histologically characterized by dilated and intermingled vessels of different sizes located in the superficial dermis, all lined by a single layer of ECs and filled with blood 22. Additionally, BRBNS frequently involves gastrointestinal VMs, which can result in consumptive coagulopathy and chronic anemia 23.

VVMs are non-hereditary and present clinically as cutaneous capillary venous malformations. They appear as well-demarcated purpuric linear plaques covered by a hyperkeratotic dermis, sometimes expanding to several centimeters in size 24. VVMs may be present at birth or develop in early childhood, with a predilection for the lower extremities. In young patients, the lesions typically display a red-blue color and a soft consistency, progressively becoming hyperkeratotic as they age 24, 25.

GVMs, a rare subtype of VM, are inherited in 38-63.8% of all cases 26. Though the precise prevalence of GVM is not well-defined, they are estimated to account for approximately 70-80% of inherited VMs 27. GVMs are characterized by numerous small, multifocal bluish-purple vascular lesions primarily affecting the skin and subcutaneous tissues. These lesions are often associated with episodes of intense, paroxysmal pain 28. Histopathologically, GVMs are defined by the presence of abnormally differentiated vascular SMCs, or glomus cells, surrounding dilated veins, which are encased by flattened, atypical ECs 28.

## 3. Genetic Heterogeneity and Molecular Mechanisms of Venous Malformation

**Figure 2:**
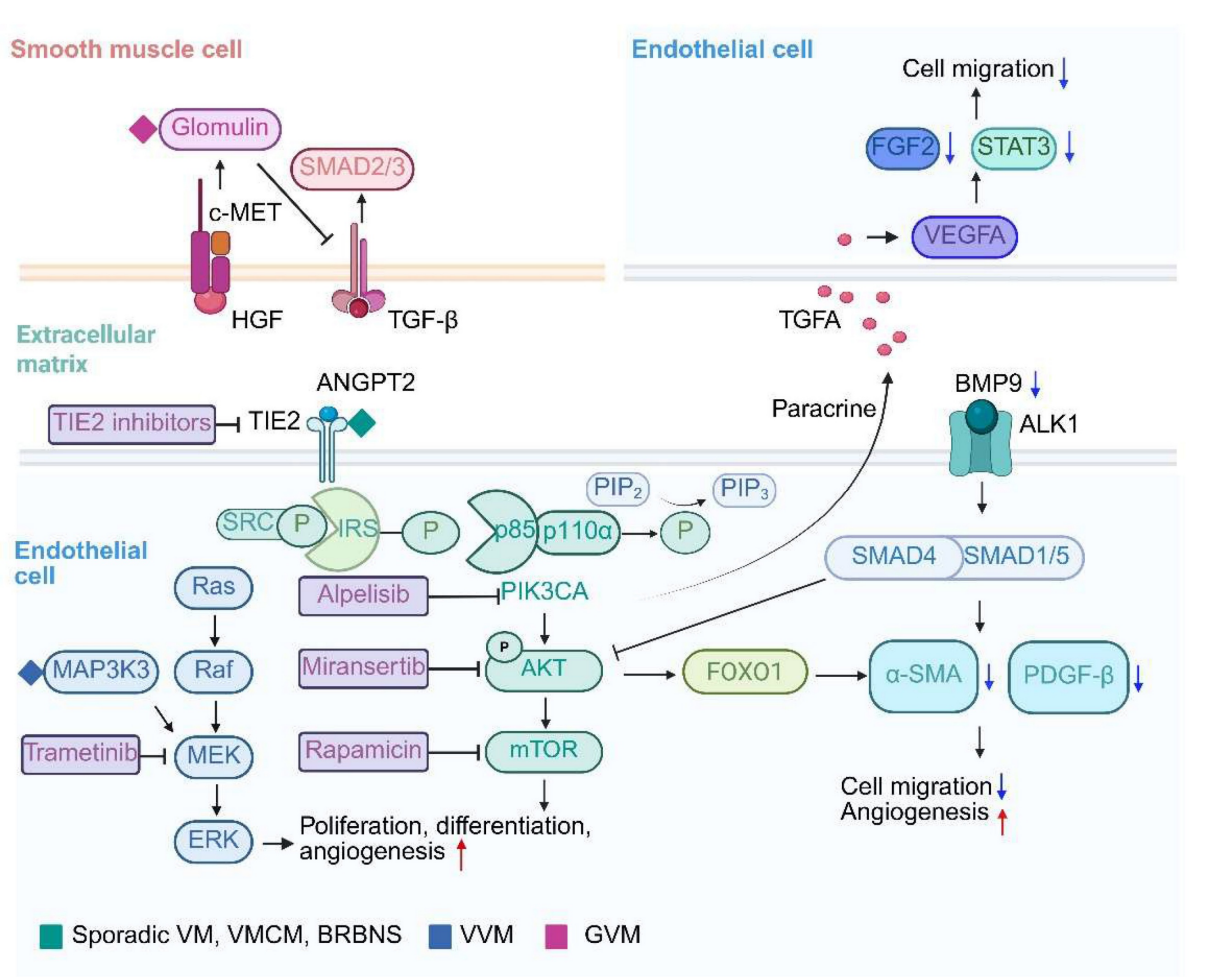
Signaling pathways and therapeutic opportunities for venous malformations. Schematic overview of PI3K/AKT/mTOR and RAS/RAF/MEK/ERK signal transduction pathways involved in venous malformations. Red and blue arrows indicate increases and decreases, respectively.

The genetic basis and pathology of VMs are highly heterogeneous, primarily due to somatic mutations in EC signaling pathways. These mutations disrupt angiogenesis, cell communication, and microenvironment homeostasis, resulting in characteristic vascular structural abnormalities (Table 1, Figure 2).

### 3.1 TIE2 signaling in venous malformations

Mutations in the *TEK* gene are the most common in VMs, present in approximately 60% of the patients 29. Located on chromosome 9q34.1, *TEK* encodes the endothelial receptor tyrosine kinase TIE2, which functions as a cell-surface receptor tyrosine kinase that is expressed in ECs 30. The angiopoietin/TIE2 kinase signaling pathway is involved in angiogenesis and lymphangiogenesis 18. In the angiopoietin/TIE system, TIE2 regulates endothelial quiescence and vascular homeostasis through spatially and temporally controlled activation 31. Angiopoietin-2 (Ang2) acts via autocrine and paracrine signaling on TIE2 to transition ECs from a stable, protective state to an activated, responsive phenotype 31. This process is critical for venous development. Gain-of-function *TEK* mutations disrupt balance by causing continuous receptor phosphorylation during critical developmental windows. These mutations overactivate the PI3K/AKT/mTOR pathway, suppressing FOXO1-mediated quiescence maintenance and impairing COUP-TFII-driven induction of venous markers like EPHB4, causing loss of venous identity 32-34. Morphologically, mutant endothelial cells exhibit aberrant proliferation due to pro-survival signaling but fail to establish luminal polarity during vascular plexus remodeling, culminating in dilated venous sinusoids devoid of smooth muscle coverage 16.

This timing-dependent pathogenesis explains VMs' distinct features: Embryonic *TEK* mutations cause multifocal, infiltrative lesions along primitive venous plexuses, while postnatal somatic mutations typically form localized lesions confined by pericyte coverage and basement membrane maturation 35.

Signaling analyses show mutant endothelial cells display arterial-like traits (like Notch target gene Hes1 upregulation) but lack arterial elastin, resulting in fragile venous walls 36. Concurrently, abnormal hemodynamic forces generate mechanical stress that perpetuates vascular wall destabilization 37. These interconnected mechanisms may form a "genetic priming-hemodynamic stress" feedback loop, driving VM lesion expansion, pain, and thrombosis via progressive endothelial dysfunction, matrix breakdown, and abnormal angiogenesis.

#### 3.1.1 Phenotypic features of *TEK*-mutant venous malformations

*TEK*-mutant VMs are more commonly observed in younger patients, particularly those under 10 years of age 38. Histologically, these VMs tend to involve the skin more frequently 38. Limaye *et al.* attributed these phenotypic differences to mutation timing and location. Unlike other VM genotypes, *TEK*-mutant VMs show higher p-AKT levels. However, no differences were found in p-mTOR or its downstream effectors (like 4EBP1 and S6K1) across VM genotypes 39. Spatial transcriptomic analysis identified SP1 as a key transcription factor regulating upregulated genes specifically in *TEK*-mutant vessels, with SP1 expression in ECs correlating with p-AKT levels in *TEK*-mutant VMs but not in *PIK3CA*-mutant cases 38.

#### 3.1.2 Heterogeneity and mechanisms of *TEK* mutations in VM subtypes

##### Sporadic venous malformations

TIE2-L914F, the most common mutation detected in sporadic VMs, is responsible for approximately 85% of these lesions and is not typically observed as an inherited mutation, implying potential lethality when present in the germline 39. The remaining 20% of VM cases result from paired double mutations that consistently occur on the same allele 39.

Multifocal VMs are predominantly attributed to double (cis) mutations, involving two somatic mutations occurring on the same allele, in contrast to the typical unifocal VMs, which are primarily driven by the somatic TIE2-L914F mutation 16, 27. The development of characteristic multifocal lesions entails a second-hit mutation in the *TEK* gene. They often exhibit mosaic patterns for the initial mutation, commonly the R915C mutation, accompanied by a second-hit mutation localized in affected regions. This somatic mutation, like the initial mosaic mutation, typically results in the introduction of an additional cysteine residue in *TEK* (like Y897C) 16.

The TIE2-L914F substitution leads to constitutive, ligand-independent autophosphorylation and sustained activation of the downstream PI3K/AKT/mTOR axis, thereby promoting EC proliferation, survival, and resistance to apoptosis 39. At the cellular level, mutant ECs exhibit abnormal cytoskeletal organization, defective intercellular junctions, and increased permeability, which translate into disorganized vessel morphology with enlarged, thin-walled venous channels lacking proper mural cell coverage 39. R915C mutation, accompanied by a second-hit mutation Y897C, also occurs within the kinase domain as an activating mutation, and shares common biological consequences with L914F, including activation of the downstream PI3K/AKT/mTOR signaling pathway and enhanced ECs activity 39.

HUVECs with the TIE2-L914F mutation can induce the formation of enlarged channels lacking pericyte/SMC coverage like VM. Specifically, primary HUVECs expressing TIE2-L914F exhibit reduced PDGFB and α-smooth muscle actin (α-SMA) levels compared to normal veins. PDGFB is crucial for pericyte recruitment as it functions as a chemoattractant for these cells, and mutations in *TEK* significantly impair the ability of ECs to produce PDGFB. This reduction is dependent on AKT phosphorylation, mediated by forkhead box O1 (FOXO1) 34. Additionally, mutant ECs inhibit SMC migration and promote the transition of SMCs from a contractile to a synthetic phenotype. This partly elucidates the reduced paracrine interaction between ECs and SMCs, and the relative lack of SMCs observed on histology of VMs 27, 34. These findings underscore the role of the AKT-mTOR/FOXO1 pathway in the interaction between mutant ECs and SMCs, contributing to venous dysmorphogenesis.

Compensatory mechanisms and therapeutic targets are emerging from pathway analyses. Li *et al.* revealed that serum and tissue levels of bone morphogenetic protein 9 (BMP9) were significantly lower in VM patients compared to healthy individuals 40. BMP9, via the activin receptor-like kinase 1 (ALK1) receptor, inhibits EC migration and enhances tube formation. Moreover, BMP9 activates the SMAD1/5-ID1 pathway, increasing the expression of structural proteins α-SMA and Desmin in SMCs, which strengthens vessel walls and maintains endothelial quiescence. In both zebrafish and mouse models, BMP9 inhibited VM development, suggesting it may reinforce the vessel wall and prevent VM formation 40. On the other hand, Chen *et al.* discovered that the enzyme UDP-glucose ceramide glucosyltransferase (UGCG), involved in the first step of glycosphingolipid synthesis, is highly expressed in TIE2-L914F mutant HUVECs. This enzyme inhibits the proliferation, migration, and tube formation of these ECs by regulating the AKT/mTOR signaling pathway, providing a potential mechanism underlying VM pathogenesis 41. Galectin-3 (Gal-3) is a member of the lectin family, upregulation of which in ocular venous malformations correlated with increased lesion invasiveness 42. Pingyangmycin treatment reduced Gal-3 expression and lesion size, suggesting that targeting Gal-3 may offer therapeutic benefits in managing aggressive venous malformations 42.

Recent advances in VMs modeling have explained critical mechanisms underlying TIE2-L914F mutation-driven pathology. Cai *et al.* demonstrated that TIE2-L914F mutant cells cultured in a three-dimensional fibrin-matrix model autonomously form enlarged luminal structures resembling human VM lesions, independent of exogenous growth factor stimulation. These aberrant vascular channels exhibited disrupted apico-basal polarity, characterized by dysregulated expression patterns of Podocalyxin and Collagen IV 43. Complementing these findings, Lazovic *et al.* established a novel TIE2-L914F induced pluripotent stem cell (iPSC)-derived endothelial cell (iEC) model, revealing that mutant iECs display upregulated migratory capacity with unaffected proliferation rates, along with dysregulation of angiogenesis-related markers 44. Under shear stress conditions, TIE2-L914F iECs showed reduced flow-directional alignment and increased cell spreading compared to wild-type counterparts 44. Building on these models, Pan *et al.* developed a differentiation protocol to generate venous- and arterial-specific endothelial cells (iVECs/iAECs) from iPSCs 45. Their work demonstrated that VM phenotypes were exclusively recapitulated in TIE2-mutant iVECs, both *in vitro* and *in vivo*, while iAECs remained unaffected. Through integration of deep learning-based drug efficacy prediction systems with digital RNA sequencing perturbation analyses, they identified bosutinib as a promising therapeutic candidate for VM treatment 45.

Fluid shear stress, a key biomechanical regulator of vascular microenvironment homeostasis, has been implicated in the pathogenesis of multiple VAs, including arteriovenous malformations (AVMs) and hereditary hemorrhagic telangiectasia (HHT) 46, 47. Ansarizadeh *et al.* employed a microfluidic vascular chip platform to study mechanobiological defects in VMs. Their findings suggest that TIE2-L914F mutations induce constitutive PI3K-AKT signaling activation, potentially impairing endothelial mechanosensing and polarization 48. Mutant cells exhibited abnormal responses to bidirectional flow patterns, mirroring the disorganized vascular architectures observed in VM lesions. Notably, comparative analyses revealed significant differences between HUVEC and iPSC-derived models: iEC mutants showed lower basal AKT activity, potentially reflecting endogenous expression levels, and displayed mechanoresponsive properties resembling immature developmental-stage endothelial cells 48.

These works highlight the critical interplay between genetic mutations, mechanical signaling, and endothelial maturation states in VM pathogenesis, while establishing innovative models for therapeutic discovery. The observed discrepancies between primary and iPSC-derived endothelial models underscore the importance of developmental context in studying vascular malformations.

##### Familial cutaneomucosal venous malformation

The most common mutation in VMCM is TIE2-R849W, transmitted in an autosomal dominant manner with incomplete penetrance, and causes weak autophosphorylation of *TEK* when overexpressed in HUVECs and needs a somatic second-hit in *TEK* to cause the typical multifocal and small-sized lesions 49. Notably, two germline substitutions, R849W and Y897S, have been identified as major contributors to these malformations 21.

As the most common mutation in VMCM, TIE2-R849W has been shown to increase phosphorylation rates and enhance cooperativity 50. The TIE2-R849W mutation specifically increases signal transducer and activator of transcription (STAT) activation. Phosphorylated STAT1 translocates to the nucleus via PI3K and MAPK-p38 pathways. This mutation uniquely induces aberrant STAT1 phosphorylation while suppressing endogenous TIE2 and STAT3 promoters. Consequently, VEGF-A-mediated STAT3 phosphorylation and FGF2 expression are inhibited, impairing endothelial cell migration and contributing to VM pathogenesis 51. Du *et al.* developed a zebrafish model by introducing transgenic expression of TIE2-R849W, providing insights into the role of epidermal growth factor-like domain 7 (EGFL7) as a potential contributor to venous abnormalities. Their research further suggests that the WNT signaling pathway could play a critical role in the development of multiple malformations, particularly in the head region 52.

##### Blue rubber bleb nevus syndrome

Soblet *et al.* detected somatic mutations in the *TEK* gene in 15 out of 17 patients diagnosed with BRBNS 23. BRBNS is specifically associated with a typical carboxy-terminal double-mutation T1105N-T1106P but also sometimes with Y897F-R915L, which is specific for multifocal sporadic VMs 23, 27. Interestingly, identical double mutations have been identified in distant lesions, while blood samples remain negative. This indicates a transiently limited circulation of mutant cells, which may instigate the formation and development of new lesions 17. Both types of mutations cause TIE2 ligand-independent activation and increase ECs' survival, invasion, and colony formation when expressed in HUVECs 23.

### 3.2 PI3K-AKT-mTOR signaling in venous malformations

*PIK3CA* (Phosphatidylinositol 4,5-Bisphosphate 3-Kinase Catalytic Subunit Alpha) gene mutations are present in almost 20% of all VMs 5. Positioned on chromosome 3q26.3, *PIK3CA* encodes the p110α catalytic subunit of PI3K, which operates as a downstream effector of TIE2 and is a vital component of the PIK3 signaling pathway 53, 54. *PIK3CA*-driven lesions are characterized by large areas of hemorrhage, hyperplastic vessels, infiltration of inflammatory cells, and increased EC density 55.

#### 3.2.1 Phenotypic features of *PIK3CA*-Mutant venous malformations

Compared to *TEK*-mutated or normal vessels, *PIK3CA*-mutated vessels downregulate genes involved in blood vessel development, positive regulation of cell migration, and extracellular matrix organization 38. However, all VM genotypes show no differences in vessel structure metrics (diameter, density, vascular smooth muscle thickness) or across different *TEK* mutation subtypes 38. *PIK3CA*-mutant and other-mutant VMs occasionally exhibit lymphocytic aggregates, whereas *TEK*-mutant VMs do not, indicating that *PIK3CA* mutations in ECs may promote lymphocytic infiltration 38.

#### 3.2.2 Heterogeneity and mechanisms of *PIK3CA* mutations in venous malformation subtypes

Activating somatic mutations in the *PIK3CA* gene located on chromosome 3q26.32 were identified in a minority of sporadic VMs 18. Among these, the p.H1047R mutation is the most frequent, followed by p.E542K and p.E545K 17.

Like *TEK* mutations, *PIK3CA* mutations activate the AKT signaling pathway. Zerbib *et al.* determined that *PIK3CA* signals predominantly through AKT1, rather than AKT2, in venous ECs, and suggested that PI3Kα may also signal through AKT-independent pathways 56.

Functionally, *PIK3CA* mutations induce a paradoxical state of hyperproliferation and premature senescence in ECs. *In vitro* studies showed that HUVECs expressing *PIK3CA* mutants lose their typical cobblestone-like monolayer structure and exhibit extracellular matrix fibronectin depletion, promoting angiogenic sprouting 57. Di Blasio *et al.* showed that *PIK3CA* mutations in VM ECs induce both hyperproliferation and cellular senescence. ECs with *PIK3CA* mutations exhibit increased β-galactosidase activity and enlarged cell size, a hallmark of senescence 55.

*PIK3CA*-H1047R mutations also increase transforming growth factor alpha (TGFA) expression, which enriches the hypoxia signaling pathway. In a mouse xenograft model, this mutation increased lesion size and vascularization, with TGFA-induced VEGF-A secretion promoting EC proliferation via paracrine signaling 58.

Castillo *et al.* developed a mouse model expressing mosaic *PIK3CA*-H1047R, a constitutively active PI3K isoform, which faithfully replicated human VM pathology. In this model, reduced pericyte coverage and decreased arteriovenous specification markers were observed, suggesting that *PIK3CA* mutations impair vessel stability and specification 59. Importantly, inhibition of PI3K/mTOR signaling led to regression of the VM lesions in this model 55, 59. Remarkably, Everolimus, an mTOR inhibitor, normalized the proliferation rate of ECs expressing *PIK3CA* mutations but was less effective at reducing senescence. Conversely, the PI3K/mTOR inhibitor BEZ235 eliminated senescent cells by inducing apoptosis 55, 60. These findings establish *PIK3CA* mutations as central drivers of VM pathogenesis, orchestrating a cascade of pro-angiogenic signalling, metabolic stress, and disrupted vascular maturation.

## 4. Additional Genetic Insights into Venous Malformations

Researchers have identified additional infrequently mutated genes in VMs and have conducted related mechanistic studies.

### 4.1 Sporadic venous malformations

Hongo *et al.* identified a somatic missense mutation, c.121G > T (p.Gly41Cys) in *GJA4*, encoding gap junction protein alpha 4 (GJA4), a transmembrane protein that is a component of gap junctions and hemichannels in the vascular system, in 96.2% orbital cavernous VM patients, suggesting GJA4 as a potential driver 61. This mutation led to EC dysfunction, such as abnormal cell morphology, lower cellular viability, and decreased tube formation. Additionally, a hyperactive hemichannel has formed, which adversely affects the viability and function of HUVECs. These phenotypes were rescued by carbenoxolone, a non-specific inhibitor of hemichannels and gap junctions 61.

*MC4R* is the most frequently mutated gene revealed by a whole-exome sequencing of orbital VM tissues, in a study by Huang *et al.*, which encodes the melanocortin 4 receptor (MC4R, a G-protein coupled receptor primarily involved in appetite regulation) 62. These mutations led to increased expression of MC4R and alterations in downstream PI3K/AKT/mTOR signaling pathways 33. *In vitro* analyses demonstrated that MC4R significantly influences EC behaviors, including proliferation, cell cycle regulation, migration, and tube formation. Furthermore, mutations in *MC4R* were found to affect downstream signaling mechanisms, including changes in cyclic adenosine monophosphate (cAMP) concentration and the expression of various PI3K/AKT/mTOR downstream genes 62. This suggests that *MC4R* mutations play a crucial role in the pathogenesis of orbital VMs by modulating angiogenic activity in ECs.

Meanwhile, through proteomics and high-concentration functional gene screening, Wang *et al.* identified *ACTA2* as the driver gene, which primarily encodes smooth muscle alpha-2 actin (ACTA2) 63, 64. Notably, lower expression levels of ACTA2 were observed in VM tissues, whereas overexpression of ACTA2 led to increased cell proliferation, migration, invasion, and angiogenic potential in ECs. In zebrafish models with ACTA2 knockdown, vascular development defects and compromised vascular integrity were evident, alongside malformations in microvessels. The depletion of ACTA2 inhibited key signaling pathways, including Dll4/Notch1 and Ephrin-B2, while activating the Hedgehog pathway 63.

### 4.2 Verrucous venous malformations

Verrucous venous malformations are caused by activating somatic mutations in the *MAP3K3* gene (OMIM 602539), located on chromosome 17q23.3. This gene encodes mitogen-activated protein kinase kinase kinase 3 (MAP3K3), which is involved in both the ERK and AKT/mTOR signaling pathways 24.

### 4.3 Glomuvenous malformation

Nearly all GVMs result from loss-of-function mutations in the *GLMN* gene, which is located on chromosome 1p22.1 65. The *GLMN* gene encodes glomulin, a protein expressed in both ECs and vascular SMCs 66. Inherited GVMs follow an autosomal dominant pattern and are characterized by the loss of glomulin function. Lesions form in regions where a second-hit mutation occurs, implying that the development of GVM lesions requires complete loss of glomulin function 67. The most common somatic second-hit mutation in the *GLMN* gene results from acquired uniparental isodisomy, where the normal allele is lost, and the mutated allele is duplicated in the affected lesion 67.

Brouillard *et al.* conducted an analysis involving 207 patients with GVMs, revealing *GLMN* gene mutations in 156 individuals 65. To date, 40 different mutations in *GLMN* have been identified, affecting 162 families with GVMs. The majority of these mutations were detected in patients with a positive family history (143 out of 162 cases; 88%), while only 19 cases were considered sporadic 68.

As mentioned before, the *GLMN* gene mutation is the most common somatic mutation in GVM 68. The regulation of CRLs by glomulin, encoded by *GLMN*, is responsible for ubiquitination of many proteins, including ones that are important for proper vascular development 26. Glomulin interacts with the unphosphorylated mesenchymal-epithelial transition factor (c-Met) hepatocyte growth factor (HGF) receptor 69. Following HGF binding, which mediates vascular SMC migration, glomulin is phosphorylated and released, subsequently activating p70S6K kinase, a downstream target of the PI3K pathway 69, 70. Glomulin also acts as a competitive inhibitor of cullin-RING ligases (CRLs), inhibit the E3 ubiquitin ligase activity of the CRL1 complex. This regulation of ubiquitination is crucial for the modification of numerous proteins essential for proper vascular development 71. Moreover, glomulin may interact with TGF-β signaling, which plays a vital role in vascular SMC differentiation 72. *In vitro* studies indicate that glomulin activity is influenced by its interaction with FK506 binding protein 12 (FKBP12), which can bind to the TGF-β type I receptor, thereby inhibiting TGF-β signaling 73. However, the precise relationship between these interactions and lesion progression, as well as potential pharmacological targets, remains to be elucidated.

## 5. Emerging Pathomechanism Frontiers Beyond Genomics

### 5.1 Extracellular vesicles and microenvironment remodeling

**Figure 3:**
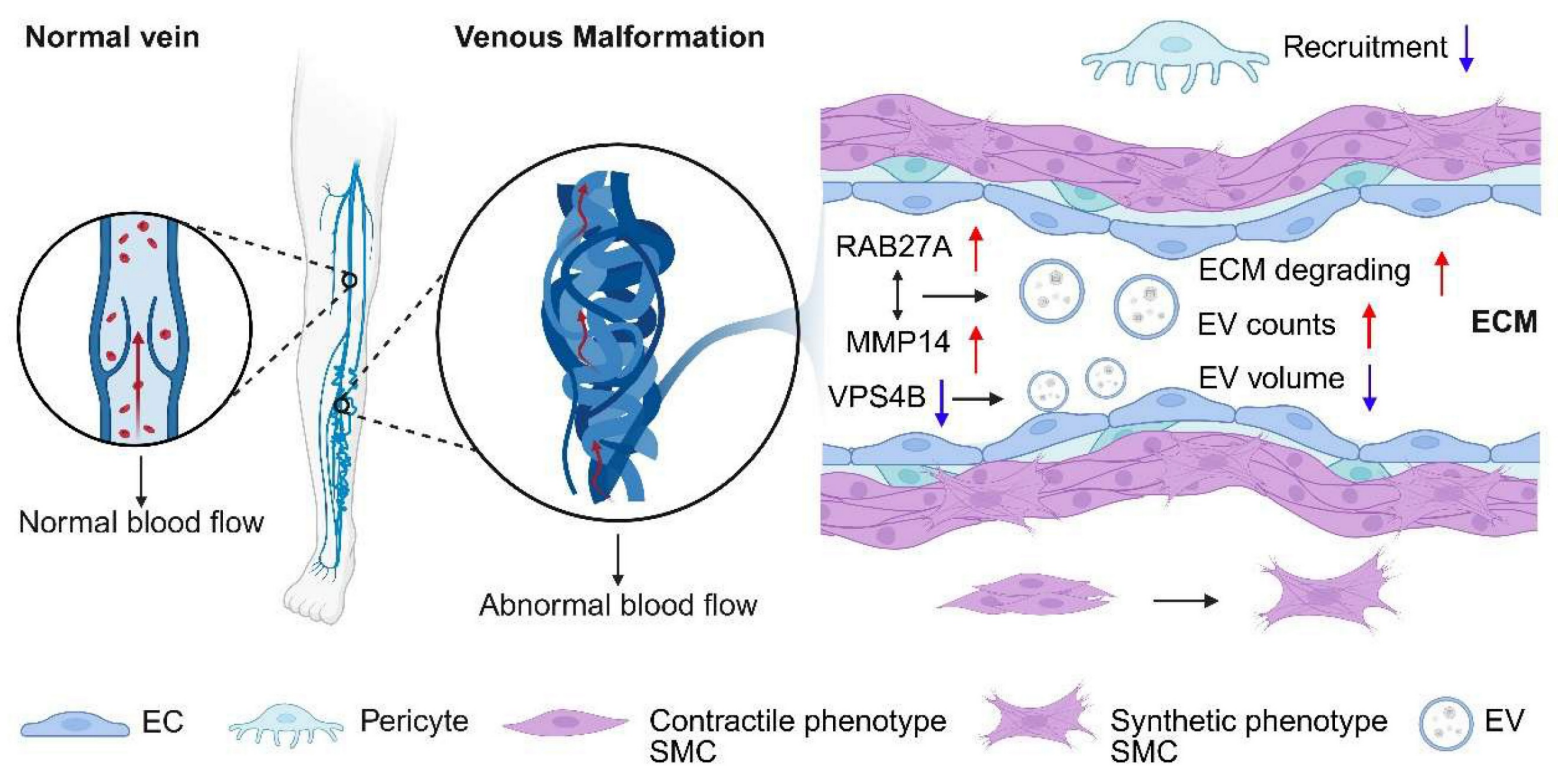
Cells and microenvironment in venous malformation tissue. In the abnormally dilated and tortuous venous channels, endothelial cells secrete an increased number of exosomes with reduced size and degrade the extracellular matrix. Pericyte recruitment is inhibited, and smooth muscle cells shift from a contractile to a synthetic phenotype.

One consistent finding across multiple investigations is the role of extracellular vesicles (EV) in mediating extracellular matrix (ECM) degradation and EC behavior (Figure 3). Chen *et al.* reported an increase in EV secretion, particularly with elevated levels of matrix metalloproteinase 14 (MMP14) in VM lesions and EC cultures harboring the TIE2-L914F mutation 74. MMP14 enrichment within EVs was linked to enhanced ECM degradation. Furthermore, RAB27A, a critical regulator of vesicle secretion, was found to positively correlate with MMP14 accumulation in the perivascular space, suggesting that RAB27A-mediated EV secretion plays a pivotal role in ECM degradation and VM progression 74.

In addition, Lai *et al.* explored the altered size distribution of small EVs in VM ECs, attributing this abnormality to reduced vacuolar protein sorting-associated protein 4B (VPS4B) expression 75. Their findings suggest that correcting aberrant AKT activation could restore VPS4B levels, normalizing EV size and pointing to a novel avenue for therapeutic interventions targeting small EVs 75.

### 5.2 Multi-omics integration and non-coding RNAs regulation

Research into VMs has identified the regulation of noncoding RNAs, intracellular signaling pathways, and vascular changes, contributing to their pathogenesis. A consistent theme across these studies is the role of "noncoding RNAs" in regulating cellular processes associated with VM development.

High-throughput sequencing analyses by Zhang *et al.* further revealed differentially expressed circRNAs, lncRNAs, and mRNAs in the serum exosomes of VM patients 76. These molecules were implicated in various cellular processes, such as protein binding, nucleic acid interactions, and cell cycle regulation. Particularly, MSTRG.9465.4 and ASAP1, which influence apoptotic pathways through the MAPK and Hippo signaling pathways, respectively, were identified as key players in VM pathogenesis 76. Angiogenic and autophagic pathways have also been implicated in VM pathology. Chai *et al.* identified upregulation of EGF and leptin in the plasma of orbital VM patients, alongside transcriptomic evidence of increased VEGF pathway activity and autophagy 77. Downregulation of pathways such as Hippo, WNT, and hedgehog further highlighted the complex regulatory environment within VM lesions. Notably, a large number of lncRNAs were differentially expressed, with E2F1 emerging as a critical transcription factor influencing their regulation 77. These results provided a novel understanding of pathogenesis and facilitated the early diagnosis of orbital VM.

Moreover, research by Yu *et al.* pointed to enriched signaling pathways, such as PI3K/AKT/mTOR and glycosaminoglycan degradation, within co-expression networks involving circRNAs, mRNAs, and ceRNA interactions in orbital VM samples 78. Interestingly, Huang *et al.* identified some common differentially expressed genes and miRNAs since infantile hemangiomas and VM share some common pathogenic factors 79.

Xia *et al.* linked the downregulation of miR-145, likely due to TGF-β suppression, to disorganized vasculature in VMs, suggesting its role as a therapeutic target in sclerotherapy 80. miR-145 was also suggested to play a role in VM sclerotherapy and represents a potential therapeutic target. Lin *et al.* found no association between the miR-618 rs2682818 C>A polymorphism and VM susceptibility 80. Similarly, Zhang *et al.* found elevated miR-18a-5p levels in VM patients, which enhanced EC proliferation and angiogenesis by modulating P53 signaling 81, while Zhu *et al.* reported miR-21 downregulation as a contributor to reduced collagen expression via the TGF-β/SMAD3/miR-21 feedback loop 82. Chen *et al.* found that VM ECs affect perivascular cells via EV-carried miR-4432, which is mediated by Ribosomal protein L36, contributing to vascular instability in VMs 83.

Additionally, lncRNA LEF1-AS1 has been identified as a significant promoter of HUVEC proliferation, migration, and angiogenesis. By acting as a competitive endogenous RNA, LEF1-AS1 binds to miR-489-3p, enhancing S100A11 expression, thus driving these cellular processes 84.

These collective findings emphasize the critical role of EV secretion, noncoding RNA regulation, and various intracellular signaling pathways in VM development. They also suggest potential diagnostic biomarkers and therapeutic targets, providing a basis for future research aimed at improving VM diagnosis and treatment.

## 6. Targeted Therapy Breakthroughs Revolutionize Venous Malformation Treatment

The initial treatment for VMs, particularly large and extensive ones, typically involves interventions such as sclerotherapy, embolization, surgical procedures, or laser ablation, which are considered the gold standard in management 85. However, these interventions are rarely curative and carry a high risk of recurrence, along with increased morbidity. Patients undergoing these treatments often experience severe chronic pain and, in some cases, significant tissue destruction, impacting their quality of life 85. Additionally, these interventions seldom achieve complete remission or are often unfeasible in most patients, primarily attributed to the extensiveness of lesions, elevated recurrence rates, and significant procedure-related comorbidity burden 17. As our understanding of the pathophysiological mechanisms underlying VMs and the involved signaling pathways advances, a new era of research has emerged, exploring the potential use of anticancer therapy in the treatment of VMs (Table 2).

The discovery of the pathogenic implication of the molecular signaling pathways in vascular malformations has facilitated the exploration of targeted pharmacotherapeutic approaches for these lesions 85. The identification of novel genes and subsequent elucidation of their functions will offer fresh insights into the etiopathogenesis of vascular pathology, potentially serving as the foundation for the development of personalized therapies that target specific mechanisms 86.

### 6.1 mTOR inhibitor

Rapamycin, also known as Sirolimus, is a selective mTOR inhibitor that disrupts mTORC1 activity, preventing the phosphorylation of downstream targets such as S6RP and 4E-BP1, both essential for cellular differentiation, proliferation, and motility 87. Initially developed as an immunosuppressant with antiangiogenic and cytostatic properties, its role in VMs has gained attention more recently 88. Preclinical studies have demonstrated the efficacy of Rapamycin and its analog, Everolimus, in slowing growth and reducing the volume of slow-flow vascular malformations by inhibiting vascular injury progression and restoring tissue vascularization 59. This mechanism involves mTORC2 disruption, specifically preventing AKT phosphorylation at SER473, which increases the activity of FOXO1 and elevates PDGFB levels, likely contributing to reduced EC proliferation 55.

The first murine VM model was established by injecting ECs expressing the TIE2 L914F mutation into mice, which produced vascular lesions resembling human VMs 29. Compared to groups injected with untreated cells or cells pretreated with TIE2 inhibitors, treatment with Rapamycin resulted in smaller and weaker vascular lesions 29. In another mouse model using the *PIK3CA-* H1047R allele, Rapamycin or Everolimus similarly delayed lesion growth and reduced lesion size 55, 59.

Multiple retrospective series have supported the clinical effectiveness of Rapamycin in both adults and children with complex, life-threatening manifestations 85. The initial dosage was 2 mg daily for adults and 0.8 mg/m² twice daily for children, with dose adjustments made to maintain target serum levels of 10-15 ng/ml 85. In the first prospective study involving six adults with symptomatic VMs, Rapamycin led to significant clinical improvements within three months, including over 50% improvement in quality of life, reduced pain, decreased D-Dimer levels, and improved coagulopathy 29. MRI scans also revealed an average 20% reduction in lesion size, with some lesions disappearing entirely. All of them experienced significant symptom relief and enhanced quality of life 29.

A subsequent phase IIB study with 19 pediatric and adult patients reported similar improvements within the first three months, along with coagulopathy resolution 89. The largest trial to date, a phase II study involving 61 patients with complex VAs, reported an 83% partial response rate after six months, which increased to 85% after one year. Most patients experienced significant quality-of-life improvements 90.

The ongoing VASE (Multicenter Phase III Study Evaluating the Efficacy and Safety of Sirolimus in Vascular Anomalies Refractory to Standard Care) trial is the largest multicenter trial to assess Rapamycin in pediatric and adult patients with slow-flow vascular malformations. Preliminary results from 101 patients treated for six months showed that 87% experienced improved quality of life, reduced pain, and alleviated functional impairment. Thirty-six patients were able to discontinue treatment after two years, though half needed to resume therapy due to symptom recurrence (EudraCT2015-001703-32; NCT02638389).

Rapamycin is generally well-tolerated, but adverse events do occur 17. A combined analysis of Phase II and VASE Phase III trials (N=122) reported an 85% incidence of toxicity, mostly mild and managed conservatively. Common side effects included fatigue, stomatitis, diarrhea, cutaneous rash, and headache. In 18% of cases, dose reduction or temporary discontinuation resolved these issues, while 10% of patients required permanent discontinuation 17, 89. Sirolimus may impair fertility and disrupt menstrual cycles, as evidenced by case reports and studies in solid organ transplant recipients, though these effects typically resolve after drug discontinuation 91. While dysmenorrhea incidence remained below 10% in the VASE trial, with resolution in all cases post-treatment, the safety profile of sirolimus in VMs warrants further clinical investigation to establish higher-grade evidence 92. Four malignancies emerged during sirolimus therapy, though the rapid onset (4-15 months post-initiation) and documented antitumor properties of sirolimus in prior studies complicate causal attribution 91.

The VASE trial demonstrated comparable therapeutic efficacy between *TEK*- and *PIK3CA*-mutated cohorts. Notably, 67% of *PIK3CA*-mutated patients exhibited improvement within the first treatment month versus 26% in *TEK*-mutated subjects. The therapeutic response emerged significantly earlier in *PIK3CA* mutations (all within 6 months) compared to *TEK* mutations (post-9 months), suggesting potential mechanistic differences in sustained mTOR inhibition 92.

Topical sirolimus (1% formulation) shows promise for cutaneous improvement while minimizing systemic toxicity, with optimal outcomes achieved over a mean period of 10.2 months 93.

Combination therapies may enhance Rapamycin's effectiveness. Co-administration of Rapamycin with the TIE2 inhibitor Ponatinib resulted in more robust inhibition of AKT, Phospholipase C gamma (PLCγ), and ERK activity, and promoted regression of highly phosphorylated ABL protein in *TEK*-mutant VMs in murine models 94. However, combining Rapamycin with pulsed dye laser therapy did not show superior efficacy 95.

### 6.2 PI3K inhibitor

Inhibition of PI3K presents a promising therapeutic strategy, with several inhibitors under clinical development. Among these, Alpelisib (BYL719) has emerged as a leading candidate, showing a favorable tolerability profile in early clinical trials 96. Alpelisib specifically targets the p110α subunit of PI3K, thereby inhibiting AKT phosphorylation 18. *In vitro* studies demonstrated that Alpelisib reduced p-AKT levels in HUVECs expressing various *PIK3CA* mutations, while also restoring normal cobblestone morphology and fibronectin expression. By contrast, rapamycin failed to normalize cell morphology or fibronectin levels. Alpelisib also inhibited AKT phosphorylation at both T308 and S473 in *TEK*-mutated ECs, suggesting its potential efficacy in treating PIK3CA- or *TEK*-mutated VAs 18, 57.

In the *PIK3CA*^H1047R^ VM model, Castel *et al.* found that Alpelisib reduced VM volume and proliferation to a degree similar to mTOR inhibitors, though it induced higher levels of apoptosis 57. A separate study using a *PIK3CA*^te2R-CreER^ mouse model, which closely mimics clinical venous malformations, compared Alpelisib, rapamycin, and miransertib. Alpelisib outperformed the other agents, demonstrating superior efficacy in reducing lesion size and preventing VMs 56.

Zerbib *et al.* further evaluated Alpelisib in 25 patients, including 7 children, with *PIK3CA*- or *TEK*-related capillary-VMs unresponsive to sirolimus, surgery, or sclerotherapy. MRI assessments showed a reduction in median VM volume by 33.4% for *PIK3CA*-related lesions and 27.8% for *TEK*-related lesions after 6 months of treatment 56. Although Alpelisib appears effective in these cases, its role in VM management remains uncertain, as most evidence comes from preclinical studies and small case series. These have shown improvements in vascular morphology, lesion size, fibronectin expression, and symptom relief 97, 98. Some studies have demonstrated comparable or superior efficacy to Rapamycin 18, 57. However, large-scale trials are needed to confirm its safety and efficacy across different VM subtypes 99.

Alpelisib exhibits a distinct adverse event profile characterized by pediatric alopecia (incidence rates reaching 30%), diarrhea (25%), hyperglycemia (13%), and growth retardation risk (23%), as recently reported by Paloma *et al.* in an oral presentation at the May 2024 ISSVA Congress, Madrid. However, unlike sirolimus, Alpelisib's status as a novel therapeutic agent precludes long-term follow-up data, particularly regarding reproductive toxicity. Furthermore, no prospective comparative studies have established alpelisib's superiority over sirolimus in treating *PIK3CA*-related overgrowth spectrum (PROS) and slow-flow vascular malformations. This evidentiary gap nevertheless positions Alpelisib as a viable salvage option for sirolimus-refractory cases 92.

Our center is conducting a study on the novel selective PI3Kα inhibitor, CYH33, for treating *PIK3CA*-related overgrowth spectrum and *PIK3CA*-associated vascular malformations. To date, 35 patients have been enrolled, including 13 with VMs. Initial results have shown promising efficacy, with detailed findings to be reported subsequently (CTR20231410).

### 6.3 AKT inhibitor

Miransertib (ARQ 092) is a potent, orally bioavailable, and selective allosteric inhibitor of AKT, currently in development for cancer and Proteus syndrome. It effectively inhibits the membrane-bound active form of AKT and prevents its activation 100.

In preclinical models, Miransertib promoted regression of proliferating VM lesions in *PIK3CA*-mutant ECs, even at low doses, and restored wild-type EC characteristics 101. Researchers found that Miransertib provided transient improvement in a patient with congenital lipomatous overgrowth with vascular anomalies epidermal nevi and scoliosis (CLOVES) and another patient with facial infiltrating lipomatosis and hemimegalencephaly 102. Encouragingly, Miransertib has been evaluated in a phase I/II clinical trial for patients with PROS and Proteus syndrome (MOSAIC study; NCT03094832). A total of 23 participants (46.9%) experienced drug-related adverse events; however, none of these events led to early study discontinuation or death. Therefore, Miransertib was considered safe and well tolerated in this patient cohort 103.

Another selective AKT inhibitor, MK2206, targets all three AKT isoforms (AKT1, AKT2, AKT3). *In vitro* studies have shown that MK2206 significantly reduces p-AKT levels, decreases FOXO1 phosphorylation, and increases PDGFB secretion in *TIE2*-mutated HUVECs 104.

### 6.4 TIE2 inhibitor and Ang-Based Clinical Prospects

Ponatinib, an Abelson kinase and TIE2 inhibitor, has been shown to inhibit the proliferation of HUVEC-TIE2-L914F cells and prevent VM lesion growth in mouse models injected with these cells 94. As previously noted, combining Ponatinib with Rapamycin produced better outcomes in VM regression compared to monotherapy in a xenograft model 94. In a refractory case involving failed sclerotherapy, laser treatment, and sirolimus, Triana *et al.* turned to targeted therapy due to life-threatening complications and therapeutic failure 105. They explored Rebastinib (DCC-2036), a potent and selective TIE2 kinase inhibitor known for reducing TIE2-positive macrophages, decreasing tumor vascular density, inhibiting tumor growth, and improving survival in murine cancer models 106. The patient exhibited notable improvements, including lesion size reduction, decreased venous dilatations, color lightening, and functional enhancements in speech and swallowing 105.

Despite these advances, challenges remain. Current TIE2 inhibitors are broad-spectrum rather than highly selective, and their efficacy has yet to meet expectations. Further research into more specific TIE2 inhibitors is necessary to optimize treatment outcomes in VMs.

Targeting Ang ligands upstream of TIE2 is theoretically attractive. Under pathological conditions, the sustained upregulation and activation of pro-angiogenic destabilizing factor Ang2 critically promote disease progression through competitive antagonism of Ang1-mediated vascular stabilization signals, inducing endothelial inflammatory responses, and promotion of vascular leakage 107, 108. Thus, targeting pathological angiopoietins (particularly Ang2) fundamentally blocks their dysregulation of the TIE2 signaling pathway. Amgen pioneered the introduction of the Ang2/Ang1 bispecific inhibitor AMG386 (Trebananib) into clinical trials; however, the agent underperformed across three phase III trials in advanced ovarian cancer-failing to show overall survival (OS) benefit and progression-free survival (PFS) benefit in the second-line treatment setting (TRINOVA-1 and TRINOVA-2 trials), and showing neither PFS nor overall OS advantage in the first-line treatment setting (TRINOVA-3 trial) 109-111. Additionally, development of the Ang2-targeting agent CVX-060 was discontinued following its Phase II trial in metastatic renal cell carcinoma (NCT01441414 and NCT01441457). Despite significant anti-angiogenic and anti-tumor efficacy in preclinical models 108, 112, Ang-targeting monoclonal antibodies failed to meet primary endpoints in pivotal Phase II/III trials, both as monotherapy and in combination with standard treatments. This limited clinical efficacy, despite promising preclinical results, indicates that targeting Ang alone is likely insufficient to provide significant clinical benefit 108.

### 6.5 Repurposing of antiangiogenetic agents

Beyond established targeted therapies, the landscape of pharmacological interventions for vascular malformations is expanding to encompass a broader spectrum of agents with anti-angiogenic potential 113.

Emerging evidence supports the therapeutic potential of repurposed agents in VA, though clinical translation faces pharmacological challenges. A case report provides evidence supporting the role of angiotensin-converting enzyme inhibitors (ACEIs) in VM management, while emphasizing the necessity for dose optimization to mitigate hypotensive effects in VM patients. Preliminary data from a small single-arm clinical trial confirm the feasibility of maintaining ACEI therapy within hemodynamically stable parameters 114. Concurrently, monoclonal antibodies and VEGF-trap molecules have garnered significant attention for their application in HHT patients, targeting pathological angiogenesis through distinct molecular mechanisms 113. Marimastat, the MMP inhibitor, targeting downstream effectors of PI3K and RAS pathways, has shown potential therapeutic efficacy with reduced toxicity profiles in AVM cohorts, as evidenced by early-phase clinical investigations 115.

Despite the potential for rapid therapeutic repurposing in VAs, drug development for these disorders remains hindered by multifaceted challenges. The inherent rarity of VA, coupled with stringent eligibility criteria and exclusionary parameters in clinical trials, severely limits patient accrual, often leading to premature trial closures before target enrollment is achieved 113. Compounding these issues, the low allelic frequency of somatic driver mutations in vascular malformations frequently results in false-negative initial testing outcomes, delaying molecular confirmation and therapeutic stratification 113.

## 7. Outlook and Conclusions

Recent molecular biology advances have significantly deepened understanding of VM pathogenesis and progression. By examining signaling pathway mutations, their genes, and encoded proteins, researchers better grasp disease etiology, enabling more promising therapies. These mutations typically alter EC proliferation, differentiation, and survival. Beyond sclerotherapy and surgery, protein inhibitors targeting disease-specific pathways are emerging as leading targeted therapies for VMs. Many VM-implicated genes overlap with oncogenesis genes, suggesting potential for repurposing cancer therapies (including combination-targeted regimens) for VMs. Among these, the mTOR inhibitor sirolimus is the most extensively studied and clinically used agent. However, sirolimus and similar therapies often fail to reverse established vascular overgrowth and carry significant side effects. These limitations underscore the urgent need for novel therapeutic target-as monotherapies or combined with existing approaches-to improve VM management. Therefore, given the low variant allele fraction (VAF) in most VM cases, accurate genetic assessment is particularly critical. Mutation-specific targeted therapy represents a highly promising treatment strategy for VMs and, more broadly, for VAs.

## Figures and Tables

**Table 1:** Classification and molecular characteristics of venous malformation subtypes​

Location	Classification	Mutated gene and typical mutation	Type of mutation	Associated signaling pathway
Isolated	Sporadic venous malformation	60% *TEK* L914F 20% *PI3KCA* (H1047R, E542, E545) Others (CDC42, GJA4, ACTA2, MC4R)	Somatic GOF mutation	PI3K/AKT/mTOR
Multifocal	Multifocal venous malformation	*TEK* (double mutation Y897C-R915C)	Mosaic GOF mutation (second-hit required)	PI3K/AKT/mTOR
	Blue rubber bleb nevus syndrome	*TEK* (double mutations T1105N-T1106P and Y897-R915L)	Somatic GOF mutation	PI3K/AKT/mTOR
	Familial venous malformation cutaneo-mucosal	*TEK* R849W	Germline GOF mutation (second-hit required)	PI3K/AKT/mTOR
	Verrucous venous malformation	*MAP3K3*	Somatic mutation	RAS/RAF/MEK/MAPK
	Glomuvenous malformation	*GLMN*	Germline GOF mutation (second-hit required)	PI3K/AKT/mTOR; TGF-β

**Table 2:** Clinical trials on targeted therapies for venous malformations

Identifier	Drug	Target	Period	scale	Phase	Condition	Status and outcomes
NCT03767660	Rapamycin (sirolimus)	mTOR	2018-2022	/	/	/	/
NCT05983159	Alpelisib (BYL719)	PI3Kα	2023-2028	n=30	II	VM, LM	Not yet recruiting
NCT02509468	Rapamycin (sirolimus)	mTOR	2015-2019	n=63	II	VM, LM	53% reduction in LM volume (n = 18)
NCT04861064	Rapamycin (sirolimus)	mTOR	2022-2024	n=24	II	VM, LM	Recruiting
NCT01811667	Rapamycin (sirolimus)	mTOR	2012-2016	n=19	III	VM, LM	100% of patients with a partial response
NCT03987152	Rapamycin (sirolimus)	mTOR	2017-2021	n=75	III	VM, LM	78% of children and 58% of adults improved QOL
NCT02638389	Rapamycin (sirolimus)	mTOR	2016-2030	n=250	III	VM, LM	Recruiting, preliminary, 85% improvement in pain and functional outcome (n = 101)
NCT04921722	Rapamycin (sirolimus)	mTOR	2021-2024	n=75	IV	VM, LM	Recruiting
